# 19. The Impact of Investigational Purified Microbiome Therapeutic SER-109 on Health-Related Quality of Life (HRQoL) of Patients with Recurrent *Clostridioides difficile* Infection (rCDI) in ECOSPOR III, a Placebo-Controlled Clinical Trial

**DOI:** 10.1093/ofid/ofab466.019

**Published:** 2021-12-04

**Authors:** Elizabeth Hohmann, Paul Feuerstadt, Caterina Oneto, Charles Berenson, Christine Lee, Sissi Pham, Lei Zhu, Pat Ray Reese, Henry Wu, Elaine E Wang, Elaine E Wang, Lisa von Moltke, Kevin W Garey

**Affiliations:** 1 Massachusetts General Hospital, Boston, Massachusetts; 2 Yale University School of Medicine/PACT-Gastroenterology Center, Westport, Connecticut; 3 NYU Langone, New York, New York; 4 State University of New York at Buffalo, Buffalo, New York; 5 University of British Columbia, Victoria, British Columbia, Canada; 6 AESARA, Chapel Hill, North Carolina; 7 Aesara, Chapel Hill, North Carolina; 8 Aesara, Inc., Apex, North Carolina; 9 CR Medicon Research, Piscataway, New Jersey; 10 Seres Therapeutics, Cambridge, Massachusetts; 11 University of Houston College of Pharmacy, Houston, Texas

## Abstract

**Background:**

Following standard of care antibiotics, investigational microbiome therapeutic, SER-109, achieved superiority vs placebo (PBO) at 8 weeks in reducing rCDI in patients with ≥3 prior episodes (12.4% vs 39.8%, respectively; p< 0.001). We evaluated the impact of SER-109 vs PBO on HRQoL with general (EQ-5D-5L) and disease-specific (Cdiff32) measures [Garey 2016].

**Methods:**

EQ-5D-5L measures outcomes in 5 domains (mobility, self-care, activities, pain/discomfort, and anxiety/depression) while Cdiff32 measures outcomes in 3 domains (physical, mental, and social) including 5 associated subdomains. Patients completed EQ-5D-5L and Cdiff32 measures at baseline (BL), Wk 8, and at recurrence/early termination. Changes from BL were assessed between SER-109 vs PBO and by clinical outcome (recurrence versus nonrecurrence) in the ITT population and within each treatment arm. The between treatment group comparison analysis controlled for age, gender, prior antibiotics, and number of prior CDI episodes.

**Results:**

Mean EQ-5D-5L and Cdiff32 scores were comparable between SER-109 and PBO at BL. EQ-5D-5L did not detect differences at Wk 8 from BL between SER-109 and PBO or by clinical outcome. In contrast, Cdiff32 detected significant improvements at Wk 8 from BL within both SER-109 subjects and PBO subjects (Fig1) and by recurrence status (Fig2). Subjects achieved significant improvement in all domains at Wk 8 from BL regardless of treatment group. When examining recurrence status within treatment arms, all PBO subjects with non-recurrence showed improvement in all health domains, while PBO subjects with recurrence had declines in several subdomains (Fig3B). Similarly, SER-109 subjects with non-recurrence showed improvement in all domains compared to BL. However, overall and mental domain/subdomains scores also improved in SER-109 subjects with recurrence (Fig3A).

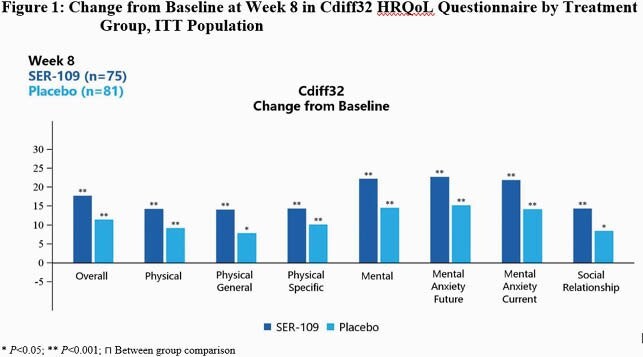

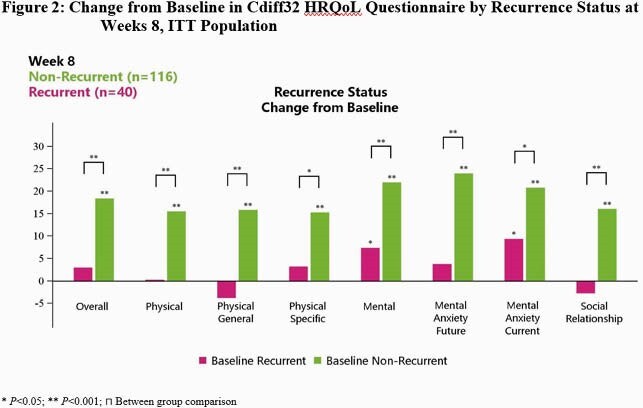

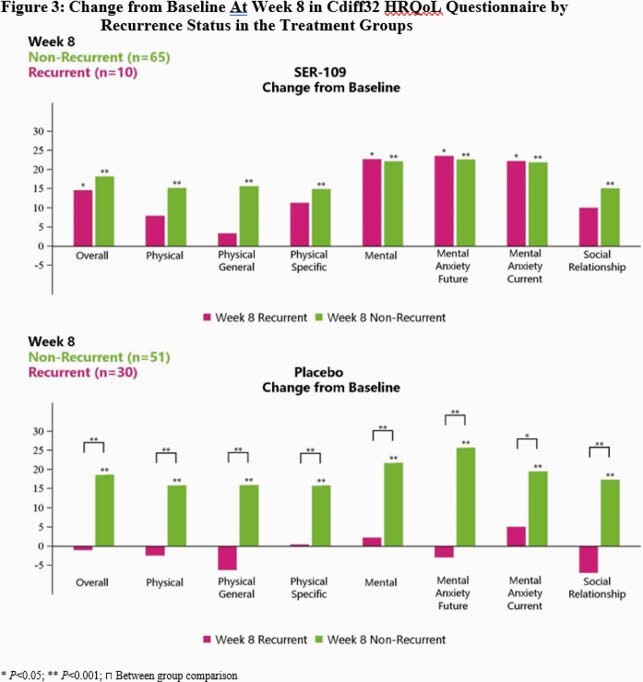

**Conclusion:**

Significant HRQoL improvements were associated with CDI nonrecurrence, which highlights the negative impact of this debilitating infection. SER-109 was associated with improved overall and mental scores, regardless of clinical outcome. Further investigation is warranted on the impact of SER-109 on mental health even among those with CDI recurrence.

**Disclosures:**

**Elizabeth Hohmann, MD**, **Seres Therapeutics** (Research Grant or Support) **Paul Feuerstadt, MD, FACG**, **Ferring/Rebiotix Pharmaceuticals** (Consultant, Scientific Research Study Investigator, Speaker’s Bureau)**Finch Pharmaceuticals** (Scientific Research Study Investigator)**Merck and Co** (Speaker’s Bureau)**SERES Therapeutics** (Consultant, Scientific Research Study Investigator)**Takeda Pharmaceuticals** (Consultant) **Christine Lee, MD, FRCPC**, **Pfizer** (Board Member)**Rebiotix-Ferring** (Board Member)**Rebiotix-Ferring** (Grant/Research Support)**Seres** (Grant/Research Support)**Summit** (Grant/Research Support) **Sissi Pham, PharmD**, **Seres** (Consultant) **Pat Ray Reese, PhD**, **Reese Associates, LLC** (Consultant, Independent Contractor) **Elaine E. Wang, MD**, **Seres Therapeutics** (Employee) **Elaine E. Wang, MD**, **Seres Therapeutics** (Employee, Shareholder) **Lisa von Moltke, MD**, **Seres Therapeutics** (Employee, Shareholder) **Kevin W. Garey, Pharm.D., M.S., FASHP**, **Summit Therapeutics** (Research Grant or Support)

